# Review on progress, challenges, and recommendations for implementing the One Health approach in the Eastern Mediterranean Region

**DOI:** 10.1016/j.onehlt.2025.101057

**Published:** 2025-05-01

**Authors:** Dalia Samhouri, Heba Mahrous, Asma Saidouni, Amgad El Kholy, Ramy Mohamed Ghazy, Mahmoud Sadek, Chiori Kodama, Elizabeth Tayler, Miriam Holm, Samira M. Al Eryani, Eva Inam Al Zein, Faisal Saeed Al-Qahtani, Mazen Malkawi

**Affiliations:** a Country Health Emergency Preparedness and International Health Regulations, WHO Health Emergency Program (WHE), WHO Regional Office for the Eastern Mediterranean, Egypt; b Public Health Consultant, WHO Health Emergency Program (WHE), WHO Regional Office for the Eastern Mediterranean, Egypt; c Tropical Health Department, High Institute of Public Health, Alexandria University, Egypt; d Family and Community Medicine Department, College of Medicine, Abha, Saudi Arabia; e Health Information and Risk Assessment, WHO Health Emergency Program (WHE), WHO Regional Office for the Eastern Mediterranean, Egypt; f Infectious Hazard Preparedness, WHO Health Emergency Program (WHE), WHO Regional Office for the Eastern Mediterranean, Egypt; g Antimicrobial Resistance, Department of Universal Health Coverage, Communicable Diseases, WHO Regional Office for the Eastern Mediterranean, Egypt; h Malaria and Vector Control, Department of Universal Health Coverage/Communicable Diseases Prevention and Control, WHO Regional Office for the Eastern Mediterranean, Egypt; i Food Safety, Department of Healthier Population, WHO Regional Office for the Eastern Mediterranean, Egypt; j Climate Change and Environmental Health, Department of Healthier Population, WHO Regional Office for the Eastern Mediterranean, Egypt

**Keywords:** One health, Eastern Mediterranean Region, Antimicrobial resistance, Zoonotic diseases, Quadripartite organizations, Environmental health

## Abstract

**Background:**

Emerging infectious Diseases have affected many Eastern Mediterranean Region (EMR) countries in the past two decades, leading to outbreaks and considerable increases in mortality rates. In addition, fragmented or destroyed health care infrastructure due to ongoing conflicts and humanitarian crises, weak governance and regulatory capacity, including poor infection prevention and control measures, and a lack of access to clean water, sanitation, and hygiene exacerbate this region's vulnerability to health threats like antimicrobial resistance (AMR). This document aimed to provide a comprehensive overview of the progress, challenges, and recommendations for implementing the One Health approach in the EMR. It highlights the interconnected health challenges at the human- animal- environment interface, such as zoonotic diseases, vector-borne diseases, AMR, food safety, environmental contamination, and climate change impacts. The document reviews advancements made at global, regional, and national levels, including initiatives by the Quadripartite organizations (WHO, FAO, WOAH, UNEP) and frameworks like the Regional One Health Framework. Challenges in implementing the One Health approach include governance, leadership, and financing issues such as political instability, inadequate data on governance mechanisms, fragmented sectors, lack of funding, and the absence of a comprehensive legal framework. Tailoring the Regional One Health framework, securing sustainable financing, and engaging key stakeholders are crucial in addressing these challenges. Building trust among different sectors is essential for collaboration, and clear communication and well-defined roles help foster that trust. Countries are sharing their experiences and challenges and learning from one another, contributing to the overall improvement of One Health implementation and operationalization in the region.

**Conclusions:**

The EMR has achieved great progress in implementing the One Health approach. This progress should be maintained and continuously monitored to ensure the preparedness of the health system to control and prevent health hazards.

## 1 Introduction

**Fig. 1:**
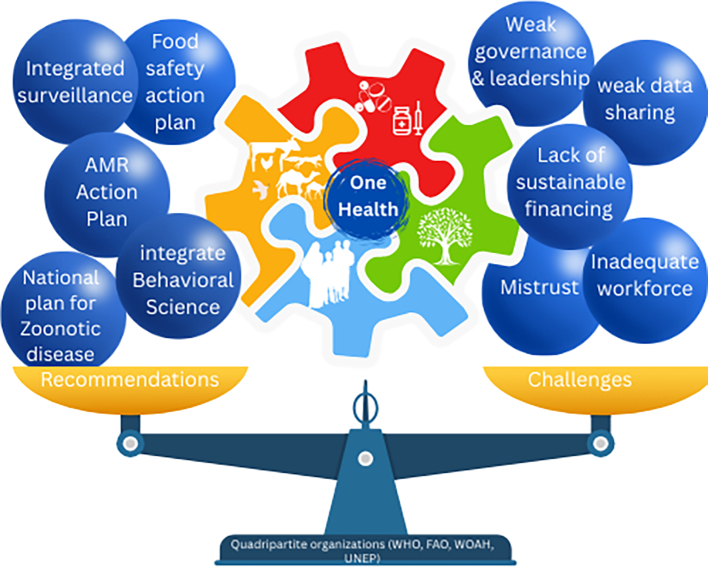
Using the One Health approach to address challenges, balance and optimize the health of people, animals, and ecosystems: components, recommendations, and barriers. [6].

The Eastern Mediterranean Region (EMR) is characterized by its high diversity and complexity, and it is particularly susceptible to emergencies caused by various hazards, including zoonotic, vector and water-borne disease. [1] Over the past two decades, zoonotic diseases have been reported in 18 of the 22 EMR countries, resulting in outbreaks and high fatality rates. Notable examples include Middle East Respiratory Syndrome Coronavirus (MERS-CoV), waterborne diseases such as cholera, arboviral infections like dengue, and the coronavirus disease 2019 (COVID-19) pandemic, which affected all countries in the region. [2,3] (See Fig. 1.)

One Health is a comprehensive and inclusive approach that strives to achieve a sustainable equilibrium and enhance the well-being of individuals, animals, and ecosystems. The approach acknowledges the interconnectedness and interdependence of human health, the health of domestic and wild animals and plants, and the broader environment, including ecosystems [4]. One Health encompasses a range of challenges including emerging, re-emerging, and endemic zoonotic diseases, neglected tropical diseases, vector-borne diseases, antimicrobial resistance (AMR), food safety and food security concerns, environmental contamination, climate change impacts, and other health risks that affect both people, animals, and the environment [5]. This review aimed to provide a comprehensive overview of the progress, challenges, and recommendations for implementing the One Health approach in the EMR. It highlights the interconnected health challenges at the human- animal- environment interface, such as zoonotic diseases, vector-borne diseases, AMR, food safety, environmental contamination, and climate change impacts.

## 2 Zoonotic diseases

Zoonotic diseases are infections that can be transmitted between animals and humans. They can be caused by various pathogens, such as bacteria, viruses, parasites, and fungi. The close interaction between humans and animals, along with factors such as habitat destruction, climate change, and changes in agricultural practices, contribute to the increased risk of zoonotic disease transmission. [7] It is estimated that over 60 % of known infectious diseases in humans have the potential to be transmitted from animals. Additionally, a significant proportion, around 75 %, of new or emerging infectious diseases originate from animals. [8] Zoonotic diseases include bacterial zoonoses (e.g., Lyme disease), viral zoonoses (e.g., avian influenza), and parasitic zoonoses (e.g., trichinosis), fungal zoonoses (e.g., ringworm), rickettsial zoonoses (e.g., Q fever), chlamydial zoonoses (e.g., psittacosis), mycoplasmal zoonoses (e.g., *Mycoplasma pneumoniae* infection), protozoal zoonoses (e.g., toxoplasmosis), and prions diseases (e.g. made cow disease) [9].

Vector-borne diseases—including malaria, dengue, leishmaniasis, Crimean-Congo hemorrhagic fever (CCHF), and other arboviruses, remain a significant public health concern in the EMR, their transmission dynamics influenced by environmental, social, and economic factors [10]. Over the past 30 years, the age-standardized prevalence and disability-adjusted life year (DALY) rates of vector-borne parasitic infectious diseases have generally declined, though with some fluctuations. The global distribution of these diseases remains highly uneven. By 2021, except for Chagas disease, the highest prevalence and disease burden were observed in low sociodemographic index (SDI) regions. Malaria had the highest age-standardized prevalence (2336.8 per 100,000 population, 95 % uncertainty interval (UI): 2122.9–2612.2) and DALY rate (806.0 per 100,000 population, 95 % UI: 318.9–1570.2). [11] Mostafavi et al., [12] reviewed the epidemiological situation of emerging and re-emerging infectious diseases in the EMR between 2001 and 2018. The study found that leishmaniasis, hepatitis A virus (HAV), and hepatitis E virus were reported in all countries, while Chikungunya, Crimean-Congo hemorrhagic fever, dengue fever, and H5N1 influenza are increasing in number and distribution. MERS-CoV remains a public health concern, and control challenges persist in some countries. Except for HAV, these diseases are either zoonotic or vector-borne infections. This emphasizes the urgent need for increased cooperation and collaboration at the human–animal–environment interface [13].

Vector-borne diseases are spreading into new regions due to a combination of environmental, social, and economic factors. Human activities such as deforestation, migration and population displacement, agriculture, energy policies, and urbanization alter vector habitats and reservoir hosts dynamics, influencing disease cycles. Climate change further impacts transmission patterns, sometimes increasing the risk of outbreaks or disrupting disease cycles. This includes the expansion of invasive malaria and dengue vectors, such as *Anopheles stephensi*, *Aedes aegypti*, and *Aedes albopictus*, increasing the risk of malaria and dengue outbreaks in previously unaffected regions. [14] [15].

Controlling vector-borne diseases can lead to significant health improvements and support economic development, especially in resource-limited settings where these diseases are both a cause and consequence of poverty [11]. A multisectoral approach for strengthened vector control involves collaboration and coordination between various sectors, such as health, environment, agriculture, and urban planning. Strengthened collaboration enables the sharing of resources, expertise, and data, leading to more efficient, sustainable, and holistic vector control strategies. [16] Current health policies often focus on individual diseases with limited coordination across sectors. However, successful programs demonstrate the benefits of intersectoral collaboration. Integrated Vector Management (IVM) is an effective approach that minimizes vector-human contact while maintaining ecosystem balance. This strategy includes targeted interventions such as habitat control, insecticide-treated bed nets, and biological agents [17]. However, some of the control strategies can lead to soil, water, and air pollution and biodiversity loss, insecticide resistance reducing the effectiveness of interventions against vector-borne diseases, and adverse acute and chronic effects on the health of populations [17,18]. The multisectoral approach also emphasizes the importance of community engagement and ative participation. Local communities are essential in preventing and implementing sustainable vector control measures, such as identifying and eliminating the breeding sites in their communities, including supporting the raising of awareness on human behavior [19].

One of the success stories is the response to viral hemorrhagic fever (VHF) outbreaks. The Regional Office for the Eastern Mediterranean of the World Health Organization (WHO-EMRO) organized a technical consultation in Tehran, Iran, in 2011. The objective of this gathering was to assess the existing gaps in the prevention and control of VHF outbreaks within the Region. During the meeting, a series of recommendations were put forth, highlighting strategic public health approaches aimed at enhancing the prevention and control of VHF outbreaks. These approaches emphasize the importance of fostering effective collaboration between the human and animal health sectors, particularly in areas related to improved preparedness, early detection, and rapid response. The successful implementation of these proposed approaches necessitates a collective effort marked by vision, commitment, and a shared sense of purpose. It underscores the significance of forging partnerships and fostering cooperation among all relevant sectors to address this critical public health challenge. [12]

## 3 Antimicrobial resistance

AMR is the ability of microorganisms—including bacteria, viruses, fungi, and parasites—to survive and continue to multiply despite exposure to drugs intended to eliminate them [20]. The improper and unrestricted prescription and utilization of antibiotics contribute to the emergence and spread of AMR, rendering the effective treatment of infectious diseases more challenging. This not only poses a direct threat to human health but also leads to increased healthcare costs, prolonged hospital stays, disabilities, and higher mortality rates [20]. National-level, high-quality data on levels of resistance and antimicrobial consumption are frequently absent as most countries lack Integrated surveillance systems. Nevertheless, available published data indicate that this issue poses a significant threat to public health in the Region, with modelled data suggesting that in 2019 there were 115,000 deaths directly attributable to AMR [21]. Research analyzed data from 2017 to 2019 in the EMR, including bloodstream infections reported to the Global Antimicrobial Resistance Surveillance System, national surveys on antimicrobial prescriptions from seven countries, and two regional surveys. The findings highlighted alarmingly high resistance rates, particularly for carbapenem-resistant Acinetobacter spp. (with an incidence of 70.3 %) and carbapenem-resistant *Escherichia coli* (with an incidence of 4.6 %) [22].

In the EMR, the widespread misuse of antibiotics and self-medication, uncontrolled sales of antibiotics, and insufficient infection control practices in healthcare facilities, along with inadequate sanitation and hygiene protocols in communities, played a role in the spread of AMR, especially in countries like Egypt, Pakistan, and Iran [[23], [24], [25], [26]]. The lack of regulation and enforcement capacity results in the inappropriate and excessive use of antibiotics in the healthcare sector.

The environmental aspect of AMR has received less attention compared with AMR in human and animal health. However, it is crucial to recognize that the natural environment serves as a significant reservoir for AMR. [27] Drug-resistant microorganisms exist in humans, animals, food, and the environment, including water, soil, and air, and the development and spread of resistance is exacerbated by inadequate pollution controls. [28] In addition to the healthcare sector, antimicrobial agents are used in plant health, particularly in crop agriculture, to control bacterial infections and ensure crop productivity and survival. They are also widely utilized in veterinary practices and food production. However, the understanding of their environmental impact and their role in the development of AMR remains limited. [29] Moreover, the COVID-19 pandemic has significantly exacerbated the burden of AMR in the region [24]. A study conducted in Egypt analyzed 2430 culture results from 2019 and 2022, revealing increased resistance in *Klebsiella pneumoniae* (*K. pneumoniae*), *Escherichia coli*, and *Acinetobacter baumannii* (*A. baumannii*). Multidrug-resistant *K. pneumoniae* and *A. baumannii* rose from 67 % to 94 % and 79 % to 98 %, while extended drug-resistant *K. pneumoniae* and *A. baumannii* increased from 6 % to 46 % and 47 % to 69 %, respectively. [30]

The implementation of Antimicrobial Stewardship (AMS) involves coordinated efforts aimed at optimizing the appropriate use of antimicrobials. While all 22 countries in the EMR have developed national action plans to address AMR, the level of implementation of these plans varies greatly among countries. Lack of funding, human resources, and technical capacity are among the key challenges in turning plans into action. Several countries in the region, including Jordan [31], Iran [32], Kuwait [33], Saudi Arabia, Oman, Qatar [34], have settled AMS to improve and evaluate the appropriate use of antimicrobial drugs.

## 4 Environmental contamination, climate change

Environmental factors such as air, water, and soil pollution, chemical exposures, and climate change play a role in over 100 diseases and injuries. Climate change, for instance, leads to fluctuations in temperature, rainfall patterns, and the occurrence of climate-related disasters, which impact the transmission of diseases among the global population. This phenomenon forces pathogens and vectors to adapt, facilitating the spread of infections and increasing the likelihood of waterborne disease transmission, especially in regions experiencing higher rainfall and flooding. [35]

The mean temperature is increasing rapidly, and other climatic variables are worsening; in the EMR in 2022, the increase reached the alarming level of 1.84 °C above the pre-industrial average. There is a 98 % likelihood that the next five years will be the warmest on record. Although the Region emits only 8.25 % of the world's greenhouse gases, its temperatures and other climatic hazards are changing twice as fast as in the rest of the world. Climatic hazards are fueling environmental degradation, natural disasters, weather extremes, food and water insecurity, economic disruption, and conflicts. The consequences for health are substantial and include more deadly extreme weather events, increasing cases of noncommunicable diseases [36], and increased emergence and spread of infectious diseases [37]. This is already impacting the Region's health workforce and infrastructure, reducing capacity to achieve universal health coverage. [38] Moreover, extreme weather events hurt food systems, resulting in the spread of both existing and emerging new foodborne pathogens, leading to environmental and food contamination. [39]

The WHO has introduced a regional framework for 2023–2029 aimed at developing climate-resilient and environmentally sustainable health systems. This framework emphasizes integrating health considerations into climate policies, strengthening the health sector's role in climate action, improving access to climate change funding, and enhancing the evidence base for policy-making. Achieving these objectives requires a comprehensive, multisectoral approach, including reinforcing healthcare systems, collaborating with key sectors such as energy, food, water, transport, finance, and security, and actively engaging the public to support climate adaptation efforts and promote overall health resilience [40].

## 5 Food insecurity

Some of the food safety issues associated with climate change that are likely to result in increased risks are the emergence of existing and new foodborne pathogens and parasites, an increase in the incidence of harmful algal blooms, increase in the incidence of mycotoxins in the environment. [41] Over the past decade, food safety-related public health events have become more complex due to the rapid distribution of pathogens in the global food chain, such as Shiga toxin-producing *Escherichia coli*, *Listeria monocytogenes,* and *Salmonella*. [42] Global warming also contributes to the proliferation of vector-borne diseases, particularly in underdeveloped countries. [43]

## 6 Efforts by the United Nations organizations to implement the One Health approach

### 6.1 At the global level

Significant progress has been made to accelerate the implementation of One Health, with notable achievements at the global level. The Food and Agriculture Organization of the United Nations (FAO), the World Organization for Animal Health (WOAH), and the WHO, collectively known as the Tripartite, have played a crucial role in endorsing and advancing the One Health concept. In February 2021, during the 27th Tripartite Annual Executive Meeting, the three organizations called upon the United Nations Environment Program (UNEP) to join their collaborative efforts. On 17 March 2022, the heads of the four organizations signed a Memorandum of Understanding (MoU) to formalize joint One Health collaboration to form a new Quadripartite Collaboration for One Health [44]. Subsequently, the One Health Joint Plan of Action (OHJPA) was launched by the Quadripartite in October 2022. The OH JPA, a five-year plan, focuses on supporting and expanding capacities in six action tracks. These action tracks aim to enhance One Health capacities within health systems, reduce the risk of zoonotic epidemics and pandemics, control and eliminate zoonotic, neglected tropical, and vector-borne diseases, strengthen food safety risks, management, and communication, address the challenge of AMR, and incorporate the environment into One Health considerations [45]. The next steps for the OHJPA involve the implementation, monitoring and evaluation, budgeting, and communication of the plan. These steps are crucial in ensuring the successful execution of the OHJPA, as they will help track progress, allocate resources effectively, and disseminate information about the initiatives and outcomes. By following these steps, the OHJPA aims to promote the health and well-being of humans, animals, and the environment through a comprehensive One Health approach [46].

### 6.2 At the regional level (EMR)

During the 36th FAO Regional Conference for the Near East in January 2022, a side event called “One Health and Combating Transboundary Diseases and Pests” was held at the regional level. The participants expressed their support and appreciation for FAO's initiative to establish a Regional One Health Platform in collaboration with all relevant stakeholders in the region. They also emphasized the importance of strengthening interagency collaborations among regional organizations to effectively support countries' efforts in implementing the One Health approach. This highlights the regional commitment and recognition of the significance of One Health in addressing transboundary diseases and pests in the EMR. [47] The 69th Regional Committee for the EMR, held in October 2022, endorsed the Regional One Health Framework, promoting One Health's implementation in the EMR. The committee called on Member States to institutionalize the approach, encourage multisectoral collaboration, and request a quadripartite One Health coordination mechanism. [48]

In early 2022, the Regional One Health Quadripartite Coordination Mechanism was established to provide leadership and strategic direction for advancing One Health initiatives across the EMR. Its objectives include convening stakeholders and partners, coordinating actions to support member states in implementing the Regional Operational Framework, and aligning efforts with the Global One Health Joint Plan of Action (OHJPA) in collaboration with the Global Quadripartite. The mechanism also facilitates sharing of the progress on One Health implementation and fosters discussions on the way forward.

The EMR held its inaugural One Health Quadripartite Regional Meeting in Muscat, Oman, from May 8–11, 2023. This landmark event equipped countries with tools to assess and develop multidisciplinary One Health core capacities, enabling them to more effectively prevent, detect, and respond to health threats at the human-animal-environment interface. During the meeting, the Regional One Health Framework was introduced, highlighting its alignment with the Global Quadripartite OHJPA and emphasizing its role in guiding coordinated action.

In 2022, the third inter-ministerial meeting on AMR convened ministers of health, agriculture, and the environment in Oman. Sixteen EMR member states signed the Muscat Manifesto, committing to ambitious targets to reduce inappropriate antibiotic use in human and animal health, and food production. This milestone underscores the region's commitment to advancing One Health by fostering collaboration between human and animal health authorities to combat the growing threat of AMR. Additionally, the WHO launched the 26th United Nations Climate Change Conference (COP26) Health Program in 2021 to promote climate-resilient and sustainable health systems. In 2023, the EM Regional Committee endorsed a framework to mitigate the health impacts of climate change, incorporating climate risk analysis into key health programs. These efforts reflect the region's recognition of the interconnectedness of environmental and public health challenges. Moreovere, over the past decade, governments and institutions have increasingly acknowledged the value of behavioral sciences (BeSc) in shaping effective policies. To this end, considerations were presented for integrating behavioral insights, data, and tools into national One Health plans, ensuring that interventions are informed by human behavior and societal contexts. Finally, the Regional One Health Quadripartite Taskforce was introduced to streamline and harmonize One Health efforts among the Quadripartite organizations at both regional and country levels. This task force aims to enhance coordination, address gaps, and drive collective action toward achieving One Health goals in the region.

### 6.3 At national level

Egypt, Jordan, Iraq, Lebanon, Somalia, and Tunisia have made significant strides in strengthening their multisectoral coordination mechanisms across human, animal, and environmental health sectors. Additionally, seven countries have successfully formalized functional coordination frameworks. Notably, a national bridging work program has been implemented in Bahrain, Jordan, Lebanon, Morocco, Pakistan, Somalia, and Tunisia to identify technical gaps and establish corrective measures. Joint Risk Assessments have also been conducted in Afghanistan, Egypt, Jordan, Iraq, Pakistan, Qatar, and the United Arab Emirates (UAE). These assessments have prioritized health threats and contributed to the development of One Health curricula. However, further clarification and support are needed to ensure these mechanisms are effective and aligned with their intended purposes. It is recommended that countries investigate and streamline these tools to enhance their functionality and impact.

### 6.4 Challenges in the One Health implementation in EMR and plans for improvement

#### 6.4.1 Governance, leadership, and financing for One Health

The One Health governance in the EMR faces challenges like political instability, lack of data, and inadequate funding. To address these, a tailored Regional One Health Framework should be maintained aligned with the OHJPA, and political commitment and advocacy are crucial for sustainable financing and long-term funding. To successfully implement One Health, innovative strategies and funding diversification are essential. Collaboration with various sectors, including the private sector, medical centres, hospitals, academic institutions, and communities, can secure additional funding. Fostering awareness of One Health's importance is also crucial.

#### 6.4.2 Trust among different sectors

Trust building among stakeholders is crucial for effective collaboration and continuity in One Health implementation. Trust-building is a continuous process, requiring commitment, cooperation, and advocacy. Prioritizing trust-building actions can lead to successful outcomes in addressing health challenges. Conflicting priorities, unclear communication, and unclear roles can hinder progress. The COVID-19 pandemic highlighted the need for a united approach to address health challenges, demonstrating how sectors can come together under One Health to tackle shared threats [49]. Fostering open communication, defining objectives, roles, and expectations for each sector, and establishing common terminology is crucial to bridging communication gaps and building trust. In addition, it is a must to facilitate regular dialogue and collaboration through joint initiatives, projects, and decision-making processes.

#### 6.4.3 Inadequate workforce capacity

The EMR faces challenges in developing an adequate workforce capacity for One Health implementation. These challenges include the limited availability of appropriate training programs and the absence of a comprehensive retention policy to address staff turnover. Additionally, there is insufficient involvement of key stakeholders in existing capacity-building activities. Also, there is a lack of knowledge and understanding about the One Health educational model. The field epidemiology training program (FETP) is a supervised, competency-based training program designed to enhance health professionals' skills and capacity to respond to health-related emergencies. It is accessible to a wide range of health professionals, promoting a multidisciplinary approach and collaboration. By incorporating FETP into workforce development strategies, countries in the EMR can address training gaps and enhance their capacity to respond to health challenges.

#### 6.4.4 Data and information exchange for One Health

There are limited resources, inadequate communication, and insufficient documentation and reporting, particularly concerning specific outbreaks. To establish a successful interaction between humans, animals, and the environment, it is crucial to implement strategies that involve comprehensive mapping of public health security, increased community support, and enhanced academic research. The One Health Monitoring Tool proves invaluable for identifying deficiencies and obstacles within One Health processes, as well as devising solutions and implementing the OHJPA. This self-assessment tool encompasses four key sectors: humans, animals, wildlife, and the environment. By utilizing this tool, gaps and bottlenecks can be effectively pinpointed, enabling targeted interventions and promoting a holistic approach to health management across these interconnected domains. [50] In addition, the Surveillance and Information Sharing Operational tool (SISOT) was developed by the Tripartite organizations and technical experts to support national authorities to establish or strengthen their coordinated, multisectoral integrated surveillance and information sharing for zoonotic diseases through assessing the national integrated surveillance and information sharing capacity already in place and linking users to a curated set of existing tools and resources that can help develop or improve that capacity.

#### 6.4.5 The achieved outcome

Countries in the EMR are well-informed about global and regional advancements in the field of One Health and equipped with the necessary resources to assess and strengthen One Health's capacity within their respective countries. Moreover, they are provided with a platform to share their experiences and present practical examples of One Health implementation, highlighting the challenges they encountered. The WHO-EMRO, in collaboration with the quadripartite organizations, supported several EMR countries in developing or updating their national One Health plans and operational frameworks based on the Regional One Health Operational Framework. This support focused on several areas, including establishing governance and leadership, and resource allocation for One Health, strengthening multisectoral coordination for One Health at the national and sub-national levels, developing mechanisms for timely information sharing among the One Health sectors and building the capacity of the One Health workforce, incorporating behavioral insights into One Health-related activity and reviewing and updating existing plans relevant to One Health, as necessary.

## 7 Key recommendation to achieve the required outcome

For the Regional One Health Framework to advance effectively, it is crucial to incorporate and expedite various components. These components include establishing a multisectoral structure, conducting the One Health risk assessment, prioritizing health threats of greatest concern, implementing necessary formalities and policies, developing a national One Health plan, and formulating a monitoring and evaluation mechanism advocating for One Health and allocating funds for its implementation.

### 7.1 At regional level

• To facilitate the implementation of One Health at the regional level, the establishment of a One Health Quadripartite steering committee is necessary. This committee would provide strong leadership, engage in political discussions, and offer overall guidance in alignment with the global OHJPA.
• Additionally, a coordination group and multiple technical groups should be formed to provide technical support and mobilize resources. Furthermore, the use of BeSc to identifying barriers and enablers to One Health implementation plays a crucial role in One Health. It acts as a complementary aspect by breaking down barriers between sectors, building trust, and enhancing communication.
• Exploring how BeSc approaches can complement and enhance efforts to address the interconnected health challenges faced by humans, animals, and the environment is crucial. By incorporating BeSc into One Health initiatives, a more holistic and effective approach can be taken to tackle public health issues and ensure the successful implementation of One Health principles. [51]

### 7.2 At the country level

• Improvements are needed in prioritizing coordination with ministries and all relevant sectors. This can be achieved through strong leadership and leveraging existing data to inform policy decisions. These elements are vital in promoting progress within the One Health framework.
• The integration of national plans addressing AMR, climate change, food safety, environmental health security, vector-borne and zoonotic disease is essential for the successful implementation of the One Health approach. It involves aligning these plans with the Regional One Health Framework specific to the EMR and OHJPA. By integrating national plans with these frameworks, countries can ensure consistency, coherence, and synergy in their approaches to addressing health issues at the intersection of humans, animals, and the environment.
• Understanding human behavior is an essential component in driving successful One Health initiatives. The use of BeSc in participatory approaches including bidirectional communication with providers and local stakeholders empowers communities to understand public health problems and design and evaluate interventions to address them, to further enhance the effectiveness, local ownership and sustainability of interventions. In addition, it promotes enabling environments and incentives, including appropriate measures in other policy areas, that encourage and facilitate behaviors that are beneficial to the physical and mental health of individuals as well as to the environment, and supportive of the development of healthy, safe and resilient communities.
• Community-centred interventions with improved risk communication to ensure communities are involved in all levels of discussion and implementation of One Health-related program.
• Building the climate resilience and environmental sustainability of health systems

By incorporating these components and addressing the necessary aspects, the implementation of One Health can be facilitated, leading to enhanced coordination, improved communication, and effective management of health challenges at the human-animal-environment interface.

## 8 Conclusions

Countries in the EMR have made notable progress in adopting and implementing the One Health approach, tailoring their efforts to address varying levels of capacity and context-specific challenges. Recognizing that each country faces unique barriers—ranging from resource limitations and infrastructure gaps to political and socio-economic constraints—governments and stakeholders have taken significant steps to adapt and advance One Health initiatives within their respective contexts. The WHO-EMRO, in collaboration with FAO, WOAH, and UNEP, is working to implement the One Health approach in the EMR. Efforts include providing updates on global and regional advancements, facilitating the exchange of experiences and best practices, introducing the Regional One Health Framework, and exploring ways to integrate behavioral insights, data, and tools into national One Health plans. The region has successfully raised awareness of One Health's progress and provided resources for capacity building. However, key challenges remain in operationalizing One Health, including governance and leadership, inadequate financing mechanisms, insufficient multisectoral coordination and trust, limited workforce capacity, poor data exchange, and insufficient attention to human behavior. To address these challenges, countries must identify areas for improvement, utilize existing data, map stakeholders, promote cross-sectoral coordination, integrate One Health into national plans, enhance workforce development, incorporate BeSc, ensure effective data governance, and advocate for resource mobilization. Collaborative efforts are essential to achieve the comprehensive implementation of the One Health approach.

## CRediT authorship contribution statement

**Dalia Samhouri:** Writing – review & editing, Writing – original draft, Conceptualization. **Heba Mahrous:** Writing – review & editing. **Asma Saidouni:** Writing – review & editing. **Amgad El Kholy:** Writing – review & editing. **Ramy Mohamed Ghazy:** Writing – review & editing, Writing – original draft, Conceptualization. **Mahmoud Sadek:** Writing – review & editing. **Chiori Kodama:** Writing – review & editing. **Elizabeth Tayler:** Writing – review & editing. **Miriam Holm:** Writing – review & editing. **Samira M. Al Eryani:** Writing – review & editing. **Eva Inam Al Zein:** Writing – review & editing. **Faisal Saeed Al-Qahtani:** Conceptualization. **Mazen Malkawi:** Writing – review & editing.

## Ethics statement

Formal ethical approval was not sought as research was not undertaken.

## Funding

This work was not funded.

## Declaration of competing interest

The authors have no conflicts of interest to declare.

## Data Availability

No data was used for the research described in the article.
